# Dry Fractionation in the Production of Andean Grain Protein Concentrates: Future Trends in Food Sustainability

**DOI:** 10.3390/foods15010120

**Published:** 2026-01-01

**Authors:** Edgar Mayta-Pinto, Daniela Edith Igartúa, José Martín Ramos-Diaz, Dario Marcelino Cabezas

**Affiliations:** 1 Programa de Doctorado en Ingeniería Agroindustrial—Mención Transformación Avanzada de Granos y Tubérculos Andinos, Universidad Nacional del Santa, Nuevo Chimbote 02712, Peru; 2024818006@uns.edu.pe; 2 Dirección General de Investigación, Universidad Peruana Unión, Lima 15102, Peru; 3 E. P. de Ingeniería de Industrias Alimentarias, Universidad Peruana Unión, Carretera Arequipa Km 6, Juliaca 21100, Peru; 4 Laboratorio de Investigación de Funcionalidad y Tecnología de Alimentos (LIFTA), Departamento de Ciencia y Tecnología, Universidad Nacional de Quilmes, Buenos Aires 1876, Argentina; daniela.igartua@unq.edu.ar; 5 Consejo Nacional de Investigaciones Científicas y Técnicas (CONICET), Buenos Aires 1425, Argentina; 6 Natural Resources Institute Finland (Luke), Humppilantie 7, FI-31600 Jokioinen, Finland; jose.ramosdiaz@helsinki.fi

**Keywords:** sustainable methods, vegetable protein, pseudocereals, quinoa, kiwicha, germ, embryo

## Abstract

The global demand for new ingredients and healthier food products is on the rise. Global challenges like rapid population growth, climate change, and emerging pandemics are putting a strain on food security for future generations. This makes it crucial to seek alternatives for producing nutrient-rich foods using more sustainable methods. In this context, proteins are an essential macronutrient for humanity. Plant-based proteins are becoming increasingly popular for the following reasons: their sustainability, as they have a lower environmental impact compared to animal-based proteins, provided they are consumed locally; their nutritional value, since they contain all the essential nutrients when consumed in a varied way and do not contain limiting amino acids; their potential accessibility; and the health benefits they offer. Consequently, the food industry is developing an increasing market of protein concentrates and isolates from plant sources using wet or dry methods. In particular, dry fractionation is expected to play a key role in enhancing food sustainability, as it allows protein enrichment without the use of water or energy-consuming operations. This review provides a detailed description of the application of dry fractionation method to Andean grains, with quinoa, amaranth, and kañiwa as prominent examples. The narrative review covers the essential primary processing and pretreatments, assesses the properties of the resulting fractions, and discusses their applications and future trends. This work aims to promote the development of innovative and sustainable food solutions.

## 1. Introduction

The world’s population has surpassed 8 billion and is projected to reach 10.4 billion by 2080, according to the United Nations [1]. This boom has created an urgent need to ensure that everyone has access to adequate, sustainable, and nutritious food [2]. This has generated an urgent need to increase current food production [3] and, simultaneously, optimize the management and distribution of existing resources. These efforts are crucial for addressing widespread nutritional deficiencies, especially protein-energy malnutrition, which currently affects 10% of the global population and requires urgent attention [4,5]. In response, the food industry has placed a high priority on developing protein-rich foods with excellent nutritional quality.

Growing interest in plant-based proteins is driven by a combination of nutritional needs, market trends, and environmental concerns [6,7]. The demand for purely plant-based products has surged due to a greater focus on health, changing dietary habits, and the emerging trends of veganism and vegetarianism. From an environmental perspective, plant-based foods are seen as more sustainable, as they require less water and land and produce fewer greenhouse gas emissions compared to animal products [8,9,10,11]. In this context, plant-based protein concentrates and isolates are emerging as innovative, sustainable, lower-cost, and potentially more beneficial ingredients for health [12,13]. Among the most promising options are Andean grains like quinoa (*Chenopodium quinoa*), kañiwa (*Chenopodium pallidicaule*), and kiwicha (*Amaranthus caudatus*), due to their protein content, nutritional quality, and economic value. These ancient crops from the Peruvian Andes have gained considerable interest in recent years due to their naturally gluten-free nature and exceptional nutritional value. They are a significant source of protein with a highly balanced profile of essential amino acids [14,15,16]. In recent years, research focused on Andean grains has intensified, covering areas from their production and processing to new product development [17]. They represent a prominent alternative to producing protein concentrates and isolates, which have good techno-functional properties, including high solubility and good foaming and emulsifying capabilities. These properties are attractive for the development of new food products, positioning these grains as suitable alternatives to meet future dietary needs [18], particularly in Latin America. Therefore, it is crucial to analyze the methods for obtaining protein isolates and concentrates from these grains. The focus should be on sustainable processes that preserve the nutritional and functional properties of the grain, ensuring they remain a viable and healthy alternative to traditional plant-based proteins (e.g., soy, pea, wheat).

Fractionation methods have been developed for obtaining protein concentrates, which are classified into wet, dry, and combined methods. While the wet process is considered the standard method for protein isolation, its high-water consumption and significant waste generation have prompted the search for more sustainable extraction alternatives [19,20,21]. In this context, dry fractionation stands out as an excellent option due to its minimal water requirements, scalability for industrial use, and ability to preserve the native functionality of proteins [22].

Previous review articles on dry fractionation have primarily focused on its application for obtaining proteins from pulses (e.g., kidney beans, lentils, peas) or cereal grains (e.g., barley, corn) [2,7,13,21,22,23,24,25,26,27]. However, there has been a notable lack of comprehensive reviews specifically focused on the dry fractionation method for protein production from Andean grains.

Therefore, the objective of this narrative review is to fill this gap by exploring recent advancements in applying dry fractionation to produce protein concentrates and isolates from Andean grains. The review will also analyze the primary processing methods and pretreatments applied to these grains before fractionation. Furthermore, it will assess the effects of dry fractionation on protein quality and discuss future perspectives related to food security. Ultimately, this work aims to disseminate recent progress in the field and to promote the development of innovative solutions to enhance the sustainability of our food systems.

## 2. Methodology

A literature search was conducted to identify publications relevant to this narrative review. The Scopus and Web of Science databases were used. The keywords employed were: fractionation, fraction, dry fractionation, milling fractionation, quinoa fractionation, physical fractionation, grain type (e.g., quinoa, amaranth, cañihua, *Chenopodium quinoa*, *Chenopodium pallidicaule*, pseudocereals, Andean grains, quinoa germ), and protein. Research articles and review publications from all years and languages focused on the application of the fractionation principle for component enrichment, with an emphasis on proteins, were considered. References cited in the retrieved articles were also included for greater comprehensiveness.

## 3. Dry Fractionation in Andean Grains

Dry fractionation is a mechanical process that concentrates and/or separates components like proteins, starch, fiber, or combinations of them from plant materials, especially grains and seeds, without using chemical agents, extensive water, or significant changes in temperature and pH [20]. This makes it a sustainable, energy-efficient method that preserves the native properties (structural, physicochemical, thermal, and techno-functional) of the resulting fractions [28,29]. Although it may produce ingredients with lower purity compared to wet methods, the fractions obtained through this process offer a significant advantage due to their industrial viability, sustainability, component enrichment with native properties, and presence of valuable micronutrients in the final composition [30].

**Figure 1:**
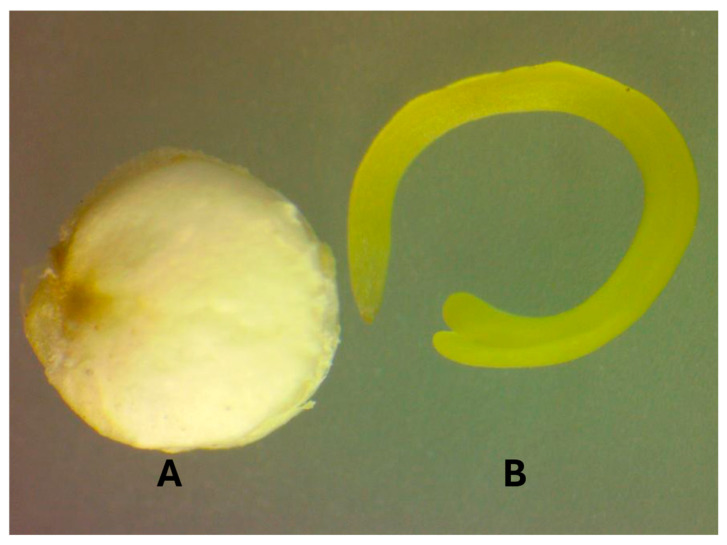
Perisperm (**A**) and germ (**B**) of the quinoa seed. Image taken with an LW SCIENTIFIC 001489 monocular microscope (40×).

Andean grains—specifically quinoa, kiwicha, and kañiwa—are excellent candidates for dry fractionation due to their unique anatomical structure and high nutritional value. These grains are notable for their high protein content and a balanced profile of essential amino acids, making them suitable raw materials for the development of high-quality protein concentrates and other ingredients (Table 1). Andean grains have very similar morphological and anatomical characteristics, structured into three main parts: perisperm, embryo, and endosperm. The perisperm is the main starch reserve, while the embryo, also known as the germ, and the endosperm are rich in protein, fat, and minerals [31]. Specifically, the embryo of quinoa and kañiwa envelops a large part of the perisperm (Figure 1) and constitutes approximately one-third of the grain. When separated from the perisperm, the germ contains around 40% protein and three times the fat content of the whole grain [32]. The kiwicha embryo represents around 25% of the grain [33,34]. Andean grains are an excellent choice for dry fractionation because a significant portion of their protein is concentrated in the embryo, which is located on the outer part of the grain. This anatomical feature facilitates the separation of the protein-rich embryo from the rest of the grain, allowing for the efficient production of high-quality protein concentrates [33,34].

**Figure 2:**
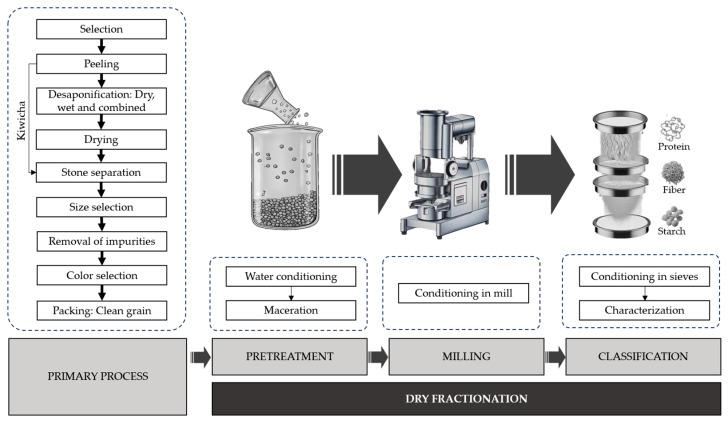
Primary processing and dry fractionation of Andean grains.

Dry fractionation is a valuable method for obtaining components from grains, but applying it to Andean grains presents significant challenges due to their unique physical properties [46]. While the basic processes include milling and sorting, the small size of these grains is the major obstacle. Quinoa seeds, for instance, are between 1.75 to 2.63 mm [47]. Kiwicha and kañiwa are even smaller, with diameters of 1.10–1.24 mm [48] and approximately 0.95 mm [49], respectively. This small size makes the milling and separation processes considerably more difficult to manage and control. Furthermore, the testa of quinoa and kañiwa grains contains a high saponin content and is closely linked to the endosperm, embryo, and the rest of the grain. The separation of these fractions influences the nutrient concentration obtained due to seed breakage, making them highly vulnerable during decortication, peeling, and polishing [33]. Given these unique characteristics, it is essential to carefully manage the primary processes and pretreatment before applying the dry fractionation method to Andean grains (Figure 2).

### 3.1. Primary Processing

The yields of fractions obtained by dry fractionation can be directly influenced by the primary processing of the grains. For quinoa and kañiwa, this initial step is critical for removing impurities and the bitter-tasting saponins present in the pericarp. Various methods have been used for de-saponification, such as dry friction, steam-drying, and water washing. While water washing is effective for saponin removal [50], it is an unsustainable method due to its high-water consumption and waste generation. Dry friction offers a more sustainable alternative, though it requires gentle mechanical treatment to avoid damaging the grain’s periphery and compromising its protein and oil content [33,51]. Methods based on steam washing and drying are considered the most suitable for removing saponin with minimal water, producing pearled grains ready for further processing [52]. Therefore, an appropriate combination of various processes, such as dry friction and steam washing, may be the key strategy to improve efficiency in grain processing plants. On the other hand, the primary processing of kiwicha is simpler, as it naturally lacks saponins and requires only cleaning and grading of the grains before further fractionation (Figure 2).

### 3.2. Pretreatment

Grains free of saponins and impurities are typically subjected to various pretreatments before dry fractionation. The main objective of these pretreatments is to optimize the yield and quality of protein fractions during milling and grading stages. Common pretreatments include adding small amounts of water (moisture conditioning), soaking, drying, hulling, and defatting [25]. Moisture conditioning, often to levels close to 14–16%, is one of the most common pretreatments (Table 2). This process makes the quinoa embryo more elastic and simultaneously softens the perisperm, facilitating the separation of both components from the seed tissues [53]. This technique has demonstrated an optimal balance between embryo yield (>84%) and embryo purity (up to 80%) [46]. Water conditioning time is also a variable to consider, as studies describe that quinoa grains with 15% moisture content for 20 h yielded a protein content of 27.78% [54]. In contrast, a shorter conditioning time of just 100 min at a similar moisture level (14–16%) reached a significantly higher protein concentration of up to 35.43% [46,55]. Similarly, moisture conditioning in kiwicha grains facilitates the separation of the testa from the endosperm during milling, thereby minimizing damage [56]. Therefore, both moisture level and conditioning time are critical variables that must be considered as pretreatments to obtain purer protein fractions with better performance from Andean grains.

### 3.3. Milling

Milling is a crucial process for obtaining protein concentrates from Andean grains using dry fractionation. It directly influences both the particle size and the overall process yield. The choice of the milling equipment and its conditions directly affect the separation, yield, and purity of the protein, starch, and fiber-rich fractions [58,67,68].

For Andean grains, the main objective of milling is to efficiently separate the protein-rich embryo from the other components, such as the starch-rich perisperm and fiber. Various mill types can be used, including pin mills, hammer mills, stone mills, disc mills, and roller mills, depending on the specific characteristics of the grain [7]. Roller milling is one of the most widely adopted methods for quinoa and kiwicha, where factors like roller gap, grain moisture content, and feed rate are critical to obtaining the desired fractions (Table 2). Due to the small size of the grains, the roller gap for quinoa must be greater than 1 mm to obtain coarse, medium, and fine fractions [57]. These fractions with different particle sizes will directly influence the chemical composition of the final products [69,70]. Furthermore, the optimal roller gap for kiwicha and kañihua should be smaller, due to the even smaller size of their grains.

### 3.4. Classification

Classification significantly influences the yield and purity of protein concentrates obtained through dry fractionation. The fraction separation process is highly dependent on particle size, as well as the classification method and parameters applied, which directly impacts the yield and efficiency of the separation. Andean grain fractions can be separated according to particle size, geometry, and density [7]. In these grains, the densest and coarsest fractions are rich in protein and fiber, while the lightest and finest fractions are primarily composed of starch (Table 2). This is a key distinction from legumes and certain seeds, where the fractional content can be the opposite [71]. This unique characteristic of Andean grains is due to their anatomical structure, where the highest concentration of protein is located in the embryo, and milling processes are specifically designed to avoid excessive particle size reduction. Most studies classify Andean grain fractions using sieves with different mesh sizes, either manually, with vibration, or by air. Air jet classification and conventional sieving are the most widely used methods and require control of factors such as mesh diameter (1, 0.800, 0.630, and 0.315 mm), which is the most influential, air flow rate (80 m^3^/h), time (2.5 min), vibration (70 Hz), and classifier speed (1500 Pa) (Table 2). Medium and coarse particle size fractions are those with the highest protein content and, therefore, also the highest lipid content [61]. This confirms that particle size directly influences the chemical composition of the fractions [60]. It has been found that medium and coarse particle size fractions contain protein levels ranging from 18.01 to 35.43% in quinoa and 17.81 to 46.6% in kiwicha [34,55,57,66] (Table 2). Studies described the achievement of protein content of up to 32.7% using air jet sieving [63]. Therefore, air classification, particularly using an air jet sieve, could be one of the suitable methods for large-scale separation, due to the difference in particle sizes between the fractions, which makes their classification relatively simple.

## 4. Effects of Dry Fractionation on Andean Grains

The dry fractionation method enables the production of protein concentrates, as well as starch and fiber, through a simplified process without additives or water usage. The resulting fractions exhibit distinct functional, chemical, and structural properties compared to whole Andean grains or flours. This is due to the concentration of nutrients, which directly influences density, water absorption capacity, solubility, and other essential properties for the food industry. The characteristics of the fractions obtained, in addition to protein content, and their functional and technological properties are described below.

### 4.1. Characteristics of the Fractions Obtained from Andean Grains

**Figure 3:**
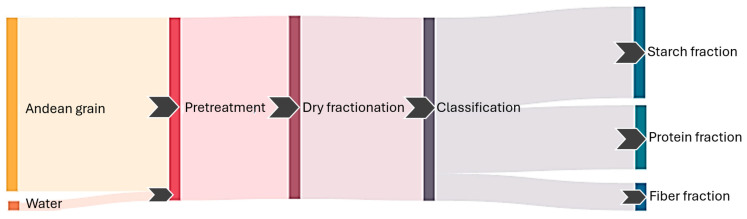
Dry fractionation in Andean grains: Steps of the process and fractions obtained.

Dry fractionation of Andean grains separates them into nutrient-rich fractions, such as protein, starch, and fiber (Figure 3). These fractions possess different compositional and functional properties, determined by particle size, density, and the morphological characteristics of the grains: germ, perisperm, and endosperm. In quinoa, the protein fraction can contain up to 35.43% protein, which would represent up to three times the protein content of the native grain. Additionally, this fraction has up to 2.74 times more fat and a higher mineral content. The fiber-rich fraction contains up to 2.35 times more fiber than the native grain, and the starch-rich fraction, with a total of 82.4%, has the lowest protein content [72]. For kiwicha, the protein content in the protein fraction can reach up to 46.6%, which represents up to 2.77 times more than the native grain protein [66]. Both the native grains and the protein concentrates obtained through dry fractionation have an excellent essential amino acid profile, with high contents of lysine (5.1–6.4%), methionine (0.4–1%), and cysteine [55]. This makes them highly valuable for nutritional applications in the food industry.

The consumption of quinoa, kañiwa, and kiwicha seeds has been linked to numerous health benefits, including antioxidant, anti-inflammatory, and anticancer properties, as well as a reduction in cardiovascular disease and improved glycemic control. These positive effects are attributed to bioactive compounds such as polyphenols, polyunsaturated fatty acids, amino acids, and fiber [73,74]. These beneficial compounds are concentrated in the fractions obtained from quinoa and kiwicha through dry fractionation. This makes these fractions even more attractive for inclusion in the diet as a grain substitute, given their enhanced nutritional characteristics. However, there is a notable gap in research, as no studies have been conducted on the application of dry fractionation to kañiwa. This is a significant missed opportunity, as kañiwa’s morphological similarity to quinoa and kiwicha suggests that dry fractionation could yield fractions with equally valuable functional properties.

### 4.2. Functional and Technological Properties of Protein Fractions Obtained by Dry Fractionation

Most current studies worldwide focus on separating and improving the yield and purity of protein fractions from Andean grains. However, information on the functional properties and technological characteristics of obtained protein-, starch-, and fiber-rich fractions is limited (Table 3). Functional properties such as water-holding capacity, oil solubility, emulsification, foaming, and pasting temperature are crucial for developing new food products, as they influence formulation, texture, and appearance. It is therefore essential to understand these properties to identify their potential applications [58].

For instance, the water-holding capacity of the protein fractions is the lowest compared to the starch and fiber fractions in both quinoa and kiwicha (Table 3), where the fiber fractions have the greatest water absorption and retention capacity [56]. High water-retaining fractions can be useful in the production of bakery products, as they influence the elasticity of the dough, gas retention, the size of the alveoli in the structure, the softness of the crumb, and stability during storge [75,76,77].

Additionally, oil retention capacity is higher in fractions with greater protein concentration (Table 3). The oil absorption medium involves capillary interaction, allowing the absorbed oil to be retained, which is related to hydrophobic proteins [55]. Therefore, this property can improve texture and flavor retention for the development of donuts, pancakes, soups, meats, and other food products [26,55].

Protein solubility is an important functional characteristic that influences emulsion, foaming, and gelation properties. This is related to the hydrophilic-hydrophobic balance of proteins and their interaction with solvents [55], as well as the dispersion of proteins in liquid media due to the presence of polar amino acids that influence hydration. These properties can be applied in the development of functional beverages, smoothies, and dietary supplements [78]. Solubility is influenced by temperature, which can reach up to approximately 18% at 93 °C in the fractions with the highest protein concentration in kiwicha and 30% at 40 °C in the protein fractions of quinoa. (Table 3).

Foaming capacity is related to the formation of a continuous cohesive protein film around air bubbles [79]. This property in quinoa fractions is influenced by pH and tends to increase under alkaline conditions (pH 12), reaching up to 19% in the starch fraction and 18% in the protein fraction, compared to the values obtained at pH 2 (Table 3). In this sense, treatments of pH shifting, combined with ultrasound, are relevant for proteins modification since they can improve foaming capacity, stability, and overall functional properties, which are crucial for developing innovative and stable food products [80,81,82]. The low foaming capacity in the protein fractions may be due to the higher fat content, which triples in the protein fractions compared to the grain [61]. The presence of fat in foaming systems can cause significant destabilization due to interfacial rupture, weakening of the stabilized protein film or its specific fatty acid profile, so it is necessary to understand the appropriate specific interactions and proportions between proteins and fat [83,84].

On the other hand, quinoa protein fractions are useful for forming food emulsions due to their high concentration of proteins with affinity for hydrophobic interfaces and their low starch content [52,61,85]. High starch content can lead to phase separation and reduced stability of emulsions since it would enhance electrostatic interactions and viscosity [86,87]. Furthermore, emulsifying capacity is influenced by temperature, increasing from 50% (25 °C) to 55% (80 °C) (Table 3), which is likely related to the partial denaturation of proteins, thus increasing their adsorption [85]. This makes these fractions suitable for the formulation of emulsified food systems, such as vegan mayonnaise, mousse-type desserts, or beverages, which could act as plant-based dairy substitutes of milk and yogurt [78].

The gelation property is the ability to form dense protein networks, creating fibrillar structures that are highly relevant in food applications due to their capacity to create desirable textures and structures [78,88]. Quinoa proteins can self-assemble, forming stable three-dimensional networks [78]. Quinoa protein fractions produced firm gel at low concentrations, starting at 12%, with gel strength increasing at higher concentrations (26%) [58]. This property can be very valuable in food industry innovation, such as the development of meat analogues and gluten-free baked goods, due to its ability to improve texture, retain moisture, enhance nutritional value, and provide structural stability [78,88,89,90].

Finally, gelatinization properties are represented by the minimum temperature required for starch gelatinization Quinoa fractions obtained by dry fractionation could present both starch and proteins, and gelatinization can be influenced by protein concentration. The data presented in Table 3 show that different fractions have unique gelatinization temperatures, ranging from 50 to 80 °C. This information can help determine the viscosity and thickening behavior of the fractions that need further study, which would facilitate the identification of potential industrial applications.

Notably, some studies conclude that the dry fractionation method better preserves the functional properties of proteins compared to fractions obtained by wet methods because only grinding processes are applied [62]. For example, coarse kiwicha fractions obtained by dry fractionation have greater swelling power and water and oil retention capacity than fractions obtained from wet methods, making them ideal for food formulations that need to maintain moisture [56]. Studies on legumes (lentils, chickpeas, broad beans, beans, peas, soybeans) also report that protein fractions by dry fractionation have better solubility compared to those from wet methods, making them more suitable in terms of functionality for food applications [91,92]. Furthermore, these grains have very similar properties to Andean grains with similar protein contents (Table 3). Therefore, the protein concentrate obtained by dry fractionation could improve the food formulation with functional characteristics, acting as a stabilizer for the development of emulsions, foams, and gels [59]. Subsequent processing, such as heat treatment or further milling, can also improve their functionality, including solubility, oil absorption, emulsification, gel strength, and even improve digestibility and amino acid content [93].

### 4.3. The Potential Applications of the Fractions

Although the application of fractions in food systems is still limited, existing studies show promising results. In extrusion, adding quinoa protein fractions (up to 25–37%) to a mixture of corn flour, rice flour, and corn starch to produce extrudates significantly increased the protein content from 7.03% to 16.20%. However, this substitution also decreased the expansion index and changed the color [94]. In bread-making, the substitution of 5–20% wheat flour by quinoa fractions rich in fiber (38.17%) and protein (21.35%) increased the protein, dietary fiber, ash, and fat contents of the blends in a dose-dependent manner. Up to 14.15, 11.12, 1.29, and 1.78%, respectively, were achieved when 20% quinoa fractions were present [95]. At the same time, the Farinograph water absorption increased whereas the dough stability decreased. The authors related these changes to gluten reduction and water redistribution induced by dietary fiber [95]. Therefore, a maximum substitution of 15% or the addition of other ingredients to increase bread volume is recommended [95]. In other applications, high-protein germ semolina and quinoa starch fractions can also be used for the development of gluten-free pastas, either alone or combined with other gluten-free ingredients, achieving protein contents between 12.2 to 21.85% [72,96,97,98]. Finally, oil can be obtained from quinoa embryo fractions via pressing, with a high content of unsaturated fatty acids, vitamin E, betalains, and carotenoids [99].

**Table 3:** Functional, technological, and thermal properties of Andean grain fractions obtained by dry fractionation.

Andean Grain	Characteristics of Fractions	Protein Content in the Fraction (%)	Water-Holding Capacity (g/g)	Oil Retention Capacity (g/g)	Solubility Index (%)	Foaming Capacity (%)	Pasting Temperature (°C)	Emulsification Capacity (%)	References
Quinoa	Protein concentrate (germ concentrate)	35.43 ± 0.15	2.78	3.10	>60 (pH 12)	The foaming capacity of germ concentrate was greater than that of other germ fractions with smaller particle sizes at pH levels: 2, 6, and 12.	--	The emulsification capacity and stability of germ concentrate were greater than those of other germ fractions with smaller particle sizes, even at different pH levels (pH levels: 2, 6, and 12).	[55]
Quinoa	Protein fraction (germ)	32.36 ± 0.23	--	--	--	--	>70	--	[52]
Quinoa	Protein fraction	33.55 ± 0.25	1.93 ± 0.13	1.15 ± 0.16	9.12 ± 0.20	8.93 (pH 2) 18.03 (pH 12)	50 ± 0.3 °C	--	[58]
	Fiber fraction	14.78 ± 0.22	3.25 ± 0.12	2.11 ± 0.11	7.17 ± 0.18	--	50 ± 0.3	--	
	Starch fraction	8.11 ± 0.26	2.13 ± 0.08	1.57 ± 0.14	7.56 ± 0.16	9.09 (pH 2) 19 (pH 12)	73.9 ± 0.4	--	
Quinoa	Protein fraction (middle fraction)	23.54 ± 1.50	1.6 ± 0.03	2.56 ± 0.17	--	5 ± 2 (mL)	79.8 ± 0.2	55.0 ± 4.0 (80 °C) 50 ± 0.0 (25 °C)	[61]
Quinoa	Protein fraction	32.7 ± 1.95 (Atlas) 32.0 ± 0.42 (Riobamba)	5 (20–60 °C)	--	Maximum solubility at 30 (40 °C) in both varieties	--	--	--	[62]
Kiwicha	Protein fraction (Coarse fraction of the seed with the highest protein content)	20.5 ± 0.5	410 ± 0.02	215 ± 0.45	17.93 ± 0.81 (30 °C)	--	--	--	[56]
Lupin	Protein content	32.7 ± 0.1	2.7 ± 0.0	--	14.9 ± 0.4	54.4 ± 0.3	--	51.6 ± 0.4	[100]
Green lentil flour	Protein content	23.13 ± 0.06	1.18 ± 0.10	0.68 ± 0.05	1.78 ± 0.00	26.11 ± 0.96	--	47.93 ± 0.27	[101]
Black-eyed beans flour	Protein content	22.10 ± 0.10	1.18 ± 0.10	0.72 ± 0.02	2.61 ± 0.00	48.89 ± 1.93	--	49.08 ± 0.46	
Pea	Protein concentrate	46.72 ±1.12	1.06 ± 0.01	1.11 ± 0.02	90.21 ± 1.85 (pH 7) 27.3 ± 1.58 (pH 3)	52.6 ± 0.02	--	66.0 ± 1.25	[102]
Pea	Protein content in flour	25.30 ± 0.98	0.94 ± 0.03	1.08 ± 0.02	70.51 ± 2.54 (pH 7)	49.20 ± 0.12	--	--	

## 5. Impact of Dry Fractionation of Andean Grains on Food Security and Sustainability

Food security, global malnutrition, and the search for resource-efficient solutions are major global challenges. For example, it is estimated that by 2024, 28% of the world’s population (2.3 billion people) lacked access to adequate nutrition, meaning they suffered from moderate or severe food insecurity [103]. In this context, plant proteins obtained through dry fractionation represent a practical, strategic, and innovative option to significantly achieve food security and sustainability [22,104], which are aligned with the Sustainable Development Goals. The application of dry fractionation to Andean grains such as quinoa and kiwicha enables the production of protein concentrate, making it valuable for addressing malnutrition issues and expanding dietary diversity [105]. Although the protein yield is lower than that of conventional wet methods, dry fractionation allows the native functionality of proteins to be maintained, and it is easily adaptable to industrial-scale processes for the development of new innovative food products.

The application of the dry fractionation method enables efficient use of resources, reduces environmental impact, and promotes a circular economy. This process does not use solvents, avoids the use of water, does not generate waste, and requires less energy compared to traditional wet methods [106,107]. Furthermore, obtaining protein concentrates from Andean grains implies a lower environmental footprint compared to proteins of animal origin, due to the use of fewer soil resources, low greenhouse gas emissions, and greater water productivity and heat tolerance [8,9,10,11]. By addressing challenges related to climate change, resource depletion, and population growth, dry fractionation becomes a model of environmental sustainability, avoiding the generation of food waste or by-products and improving environmental integrity practices [108].

Dry fractionation is currently the most sustainable method (compared to wet and combined methods) (Table 4) for concentrating protein from quinoa and kiwicha seeds, and potentially from kañiwa grains as well.

From a technical and economic perspective, no studies have been conducted on the dry fractionation method for Andean grains. However, studies on other products, such as corn, indicate that methods achieving greater purity, such as the wet method or dry fractionation with radiofrequency and infrared radiation pretreatments, require higher capital, processing, and energy costs, while also generating byproducts [112]. This makes the basic dry fractionation method the most cost-effective, as it uses small amounts of water for pretreatments [112,114].

## 6. Research and Future Perspectives

Dry fractionation of plant proteins represents a practical and strategic solution to global challenges such as food security and malnutrition. This method, particularly when applied to Andean grains, aligns with the Sustainable Development Goals by offering a path toward greater food sustainability. Optimization studies are needed to achieve higher yields and purity of protein, starch, and fiber fractions. This could include improvements in pretreatment, milling, and grading processes. For pretreatment, variables like water content, temperature, and time should be optimized to enhance yield [56]. In milling, a comparative analysis of different mill types is crucial to finding the most suitable option for the unique characteristics of each grain. For classification, dual techniques like air and electrostatic separation could be explored to improve the purity of protein fractions [25]. Additionally, emerging technologies such as laser-induced breakdown spectroscopy (LIBS) could be used for real-time structural analysis of grain tissue, thereby assessing mechanical properties and improving process efficiency [33].

Furthermore, the reported research has been conducted primarily at the laboratory level, making pilot-scale and industrial-scale studies essential. These will require new optimization studies that account for the challenges associated with upscaling. Likewise, detailed information on grain characteristics and method application can provide crucial insights for the design of specialized machinery tailored to the unique properties of Andean grains [68].

Future studies should investigate the use of these fractions in advanced food systems for the development of gluten-free products, protein supplements, and their use as functional and nutraceutical ingredients. Additionally, studies are needed on their application in the pharmaceutical, cosmetic, and industrial sectors for the development of biodegradable films. The protein, starch, and fiber-rich fractions could be used as ingredients for developing 3D and 4D printed foods, allowing the development of personalized foods to combat malnutrition. The integration of artificial intelligence (AI) can further enhance this process by predicting optimal conditions and final product quality [115,116].

Finally, a significant research gap exists for kañiwa grains, since no studies have yet reported the application of the dry fractionation to this Andean grain, which represents a research opportunity. Given its similar morphological features to quinoa and kiwicha, exploring this area represents a valuable research opportunity.

## 7. Conclusions

Dry fractionation of Andean grains is a promising and sustainable method for obtaining protein concentrates, as well as other fractions such as starch and fiber, especially when compared to other conventional grains. It also supports the revitalization of Andean crop production, which has declined drastically in recent years. This approach is essential for addressing the challenges of global food security, as it provides nutritious ingredients through an environmentally friendly process.

Unlike conventional wet fractionation, dry fractionation is a physical-mechanical process that significantly reduces the use of water and energy, while avoiding the use of chemical solvents. This aligns with the principles of a circular economy, minimizing waste and lowering the environmental impact of food production. The process is particularly well-suited for Andean grains like quinoa and kiwicha, which have their protein concentrated in the outer embryo, allowing for easier separation.

The fractions obtained from dry fractionation are not only nutritious but also retain the native functionality of the proteins, a key advantage over wet methods that can cause denaturation. This preservation of properties like solubility, emulsification, and foaming makes the resulting protein concentrates highly versatile for various food applications.

In the face of global food insecurity, malnutrition, and environmental degradation, dry fractionation of Andean grains offers a strategic path forward, enabling the development of nutritious, functional, and sustainable food ingredients while supporting local economies and global sustainability goals.

## Figures and Tables

**Table 1:** Nutritional composition (g/100 g wet basis) of Andean grains.

Andean Grain	Moisture (%)	Protein (%)	Total Carbohydrates (%)	Dietary Fiber (%)	Lipids (%)	Ash (%)	References
White Quinoa	5.1–12.0	10.0–24.0	54.0–75.3	2.1–11.9	1.5–14.6	1.5–3.7	[35,36,37,38]
Red Quinoa	9.0–9.6	12.2–20.2	71.0–75.3	9.0–16.1	5.8- 6.4	1.9–2.8	[36,37,38,39]
Black Quinoa	5.4–9.3	12.5–20.9	71.2–77.0	9.0–22.9	5.9–6.0	2.2–2.6	[36,37,38,39]
Kañiwa (Cañihua)	5.6–12.0	14.4–19.5	61.9–72.5	4.3–11.1	7.6–9.7	2.8–4.6	[38,40,41]
Kiwicha (Amaranto)	7.9–9.8	12.0–18.3	49.5–65.5	6.0–16.3	2.2–10.1	1.9–2.8	[42,43,44,45]

**Table 2:** Dry fractionation in Andean grains: Process conditions and yields.

Grain Type (% Initial Protein)	Primary Process/Pretreatment	Milling: Equipment	Milling: Parameters	Classification	Protein content in the Protein Fraction (%)	References
Quinoa (NS)	Polished and hulled/Conditioned in water for 100 min to a moisture content of 14–16%	Roller mill: Buhler Laboratory Mill (MLU-202)	NS	Manual	35.43 ± 0.15	[55]
Quinoa (15.58)	Hulling, steam washing, and drying/Water conditioning to a moisture content of 15.50%	Roller Mill: Buhler Laboratory Scale (MLU-202)	NS	Manual	32.36 ± 0.23	[52]
Quinoa (11.30)	NS	Roller Mill: E8, Haubelt Laborgeräte GmbH, Istanbul	NS	Vibrating screens	18.01	[57]
Quinoa (14.78 ± 0.22) in whole wheat flour	Cleaning and removal of foreign matter/Water conditioning to a moisture content of 14–16%	Buhler Laboratory Roller Mill (MLU-202)	NS	Manual	33.55± 0.25	[58]
Quinoa (NS) Tunkahuan Variety	NS	Continuous flow mill—MAVIMAR, Popayán	NS	Manual	31.5	[59]
Quinoa (16.09 ± 0.29)	Polished hull 8%/Conditioned with water for 100 min to reach a moisture content of 14–16%	Buhler Laboratory Roller Mill (MLU-202)	NS	Manual	34.78 ± 0.18	[46]
Quinoa (14.12)	NS/No pretreatment	Laboratory mill—Grain mill, KitchenAid, model 5KGM, Whirlpool Corporation	NS	Retsch AS 200 basic vibrating sieve (Haan)	18.86 ± 0.05	[60]
Quinoa (15.58 ± 0.94) Titicaca variety	Saponins removed by polishing/No pretreatment	Ball mill Pulverisette 6, Fritsch	NS	Manual	23.54 ± 1.50	[61]
Quinoa (14.1 ± 0.6) Riobamba variety	NS/No pretreatment	Laboratory mill—Fritsch Mill Pulverisette 14, Idar-Oberstein	Rotor speed: 4000 g, with a feed rate of ∼20 g/min	Air-jet sieving (Alpine200 LS-N, Hosokawa-Alpine, Augsburg, Germany) with various sieves (0.800, 0.630, and 0.315 mm) at 1500 Pa for 2.5 min	32.0 ± 0.3	[28]
Quinoa (NS) Atlas and Riobamba varieties	NS/No pretreatment	Laboratory mill—Fritsch Mill Pulverisette 14, Idar-Oberstein	Rotor speed: 4000 g, with a feed rate of ∼20 g/min	Air-jet sieve (Alpine200 LS-N, Hosokawa-Alpine, Augsburg, Germany) with various sieve sizes (0.800, 0.630, and 0.315 mm) at 1500 Pa for 2.5 min	32.7 ± 1.95 (Atlas) 32.0 ± 0.42 (Riobamba)	[62]
Quinoa (11.6)	NS/No pretreatment	Laboratory mill (Fritsch Mill Pulverisette 14, Idar-Oberstein, Germany) with mesh sizes of 1.5 and 2.0 mm	Rotor speed: 6000 g, with a feed rate of ∼20 g/min	Air-jet sieve (Alpine 200 LS-N, Hosokawa-Alpine, Augsburg, Germany) with various sieve sizes (1, 0.85, 0.63, 0.5, and 0.315 mm) at 1500 Pa for 2.5 min. 27.8 ± 0.0	27.8 ± 0.0	[63]
Quinoa (NS) tall varieties	NS/No pretreatment	Rotor mill (brand not specified)	Air flow rate of 40 m^3^/h and 2.0 mm sieve opening	ATP50 classifier (Hosokawa-Alpine, Augsburg, Germany) with a classifying wheel speed of 1000 rpm and an air flow rate of 80 m^3^/h.	23.5	[64]
Quinoa (11.75) Real variety from Bolivia	NS/Conditioning with water to 15% moisture content for 20 h	Roller mill: Brabender Quadrumat Junior -Duisburg	NS	Chamber sieve from Bühler GmbH (Braunschweig, Germany) with a set of graduated standard sieves	27.78 ± 1.10	[54]
Kiwicha (NS)	NS/No pretreatment	Laboratory grain mill (Kitchenaid, Whirlpool Corporation, Benton Harbor)	NS	Retsch AS 200 basic vibrating screen (Haan, Germany) for 30 min at an amplitude of 70 Hz	29.36 ± 0.01	[65]
Kiwicha (14.8 ± 0.13) variety K432	Removal of foreign matter/Water conditioning to 18% moisture content	Laboratory-scale roller mill (Buhler, Switzerland)	Feed rate: 6 kg/h. Roller gap: 0.61–0.13 mm	200 μm laboratory hand sieve	20.5 ± 0.5	[56]
Kiwicha (14.60 ± 0.361) variety K432	Removal of foreign matter/Conditioning with water to 16% moisture content for 24 h	Roller mill: (Buhler, MLU-202) Laboratory scale	NS	Manual	17.81 ± 0.26	[34]
Kiwicha (16.8 ± 0.1)	Removal of foreign matter/NS	Suzuki MT95 Laboratory Rice Mill Suzuki, São Paulo	NS	Manual	46.6 ± 0.2	[66]

NS: Not specified.

**Table 4:** Impact of dry and wet fractionation methods on protein separation.

Aspect	Dry Fractionation	Wet Fractionation	References
Environmental Impact	Lower environmental impact due to minimal use of water and chemicals.	Greater environmental impact due to intensive use of water, energy, and chemicals.	[109,110,111]
Energy Consumption	Much lower, even in mechanical processes without complex treatments.	High energy consumption during heating and drying processes.	[109,110,111]
Protein Functionality	Minimal processing. Preserves the protein’s native functionality.	Often produces denatured proteins, reducing or altering their functionality.	[110,111]
Yield and Purity	Generally, lower protein yield and purity.	Higher protein yield and purity.	[109,111]
Capital and operating costs	Capital and operating costs are typically lower in subdivisions located in dry areas, making them more attractive from an economic perspective.	Generally, they have higher capital and operating costs due to their intensive use of water and energy.	[112]
Sustainability	More sustainable due to lower resource use.	Less sustainable due to high resource consumption.	[109,110]
Challenges	Dispersibility and flowability problems with high humidity during pretreatment.	High environmental impact and loss of functionality of native proteins.	[113]

## Data Availability

No new data were created or analyzed in this study. Data sharing is not applicable to this article.
